# The afatinib resistance of *in vivo* generated H1975 lung cancer cell clones is mediated by SRC/ERBB3/c-KIT/c-MET compensatory survival signaling

**DOI:** 10.18632/oncotarget.7746

**Published:** 2016-02-26

**Authors:** Laurence Booth, Jane L. Roberts, Mehrad Tavallai, Timothy Webb, Daniel Leon, Jesse Chen, William P. McGuire, Andrew Poklepovic, Paul Dent

**Affiliations:** ^1^ Departments of Biochemistry and Molecular Biology, Virginia Commonwealth University, Richmond, VA 23298, USA; ^2^ Department of Medicine, Virginia Commonwealth University, Richmond, VA 23298, USA

**Keywords:** H1975, ERBB1 T790M L858R, afatinib resistance, dasatinib, ERBB3+c-MET+c-KIT

## Abstract

We generated afatinib resistant clones of H1975 lung cancer cells by transient exposure of established tumors to the drug and collected the re-grown tumors. Afatinib resistant H1975 clones did not exhibit any additional mutations in proto-oncogenes when compared to control clones. Afatinib resistant H1975 tumor clones expressed less PTEN than control clones and in afatinib resistant clones this correlated with increased basal SRC Y416, ERBB3 Y1289, AKT T308 and mTOR S2448 phosphorylation, decreased expression of ERBB1, ERBB2 and ERBB3 and increased total expression of c-MET, c-KIT and PDGFRβ. Afatinib resistant clones were selectively killed by knock down of [ERBB3 + c-MET + c-KIT] but not by the individual or doublet knock down combinations. The combination of the ERBB1/2/4 inhibitor afatinib with the SRC family inhibitor dasatinib killed afatinib resistant H1975 cells in a greater than additive fashion; other drugs used in combination with dasatinib such as sunitinib, crizotinib and amufatinib were less effective. [Afatinib + dasatinib] treatment profoundly inactivated ERBB3, AKT and mTOR in the H1975 afatinib resistant clones and increased ATG13 S318 phosphorylation. Knock down of ATG13, Beclin1 or eIF2α strong suppressed killing by [ERBB3 + c-MET + c-KIT] knock down, but were only modestly protective against [afatinib + dasatinib] lethality. Thus afatinib resistant H1975 NSCLC cells rely on ERBB1- and SRC-dependent hyper-activation of residual ERBB3 and elevated signaling, due to elevated protein expression, from wild type c-MET and c-KIT to remain alive. Inhibition of ERBB3 signaling via both blockade of SRC and ERBB1 results in tumor cell death.

## INTRODUCTION

It is well known that the signal transduction pathways in a tumor cell, from receptor to nucleus, exhibit a high degree of plasticity and redundancy thereby enabling the tumor cell to rapidly evolve as the tumor grows to environmental stresses such as hypoxia or nutrient depravation. Similar forms of stress-induced changes in receptor signaling and in the overall transduction from membrane to nucleus occur in tumor cells exposed to anti-cancer therapeutic modalities, including traditional DNA damaging chemotherapies and ionizing radiation. For example, many millions of dollars and person-hours have been spent in the last 25 years attempting to synthesize potent specific inhibitors of drug efflux pumps such as ABCB1 and ABCG2; pumps that are over-expressed in recurrent tumors and thus reduce the efficacy of chemotherapy [1]. More recently, with the development of drugs that have been developed as “specific” inhibitors of protein kinases we have observed a new dimension in tumor cell evolution, for example in which mutated oncogenic growth factor receptors under the selective pressure of a kinase inhibitor gain additional mutations to render themselves resistant to the original kinase inhibitor drug or where tumor cells utilize the redundancy between some survival signaling pathways, changing their need for survival signaling from one pathway to another signaling pathway [2, 3].

It is widely accepted that some tumors and tumor cell types are at initial presentation highly addicted for growth and viability to one specific mutated enzyme, usually a protein kinase but sometimes a GTP binding protein. This mutated kinase will be one-two orders of magnitude more active than its wild type variant and will, upon transfection into susceptible non-transformed cells cause transformation, most often also with a large increase in cellular tumorigenic potential. However, although some tumor cell types very frequently have variants that exhibit a specific addiction to a single kinase: e.g. BCR-ABL in chronic myelogenous leukemia; mutated active ERBB1 in non-small cell lung cancer; mutated active B-RAF in melanoma; the majority of other tumor types and subtypes have a pleiotropic combination of mutations in tumor promoters and tumor suppressors that collectively facilitate tumorigenesis: e.g. in breast cancer; head and neck cancer; liver cancer [4–6]. It is even possible, as we note in the associated companion manuscripts, that tumor material upon standard of care screening does not present with any of the known well defined mutations in proteins linked to tumorigenesis in that tumor type; e.g. the July 2015 PDX isolate of non-small cell lung cancer, ADOR.

Because of the simplicity with which an investigator can generate a durable stable disease; partial response; complete response for patients whose tumors have a sole specific addiction to the signals of one specific mutated enzyme, the cancer experimental therapeutics field has evolved over the last decade to pursue concepts in which tumors can be controlled for several years by a single drug, even when the driving oncogene may only be present in ∼2% of all patients with that particular malignancy. This has led to the creation of the “personalized medicine” concept where, by genetic screening, the physician will have a list of the cellular proto-oncogene proteins that have activating/inactivating mutations and can thus treat that patient specifically with the drug most likely to block those oncogenic signal(s). However, at present, we have neither sufficient biological understanding of pathway dynamics nor computer software to deconvolute the much more complicated everyday “*every-patient*” scenario where the patient's tumor does not contain those one or two obvious driving oncogenes. As genetic DNA/RNA screening does not reveal the regulatory protein phosphorylation levels in any protein, without an unbiased screen of protein expression and protein phosphorylation levels, such DNA/RNA based diagnostic assays have a high probability of failure.

The present studies were designed to ask whether, using traditional biochemical methods and using a minimum of DNA screening, we could define the signal transduction changes that occur when a tumor cell expressing a driving oncogene, in our case ERBB1 T790M L858R in the H1975 non-small cell lung cancer line, is made resistant to a clinically relevant standard of care drug that would be used to treat such a tumor in a patient, afatinib. We generated afatinib resistant H1975 tumor clones using *in vivo* transient exposure of established flank tumors to the drug and studied without any bias, the changes in tumor cell biology.

## RESULTS

**Figure 1:**
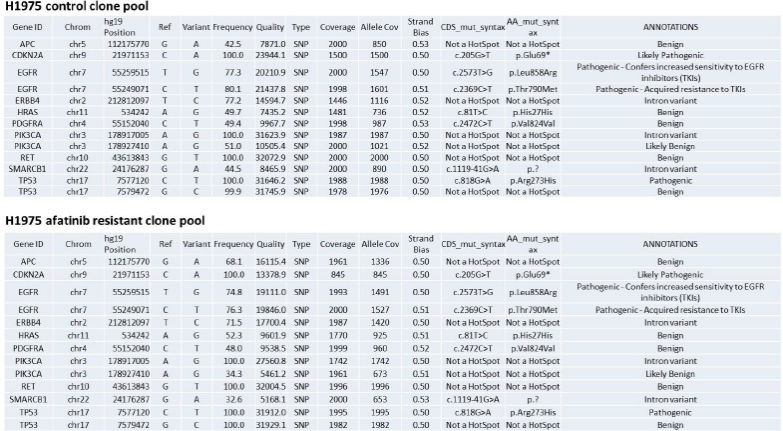
Afatinib resistant H1975 clones do not exhibit any alteration in the mutational status of well characterized proto-oncogenes Pooled control clones and pooled afatinib clones from the H1975 tumors were subjected to sequencing analyses on an Ion Ampli-Seq™ Cancer Hotspot Panel v2 screen for mutations in 50 genes, performed by the VCU Health System/Department of Pathology.

We generated by transient *in vivo* high dose afatinib treatment, five afatinib-resistant H1975 tumor clones; and in parallel five vehicle control tumor clones. H1975 non-small cell lung cancer cells express a double mutated active ERBB1 and for a patient with such a tumor, afatinib would be the standard of care treatment. Pooled control clones and afatinib resistant clones were subjected to an Ion Ampli-Seq™ Cancer Hotspot Panel v2 screen for mutations in 50 genes, performed by the VCU Health System/Department of Pathology. The results, supplied to us by The VCU/MCVH Department of Pathology, showed no mutational changes in the majority of the potential mutated sites tested (data not shown). In those proteins where mutations were discovered, mutations that could/will have biologic consequences for the cell, we discovered that no frequently observed new “hotspot” site of mutation was found in the afatinib resistant clones (Figure 1).

**Figure 2:**
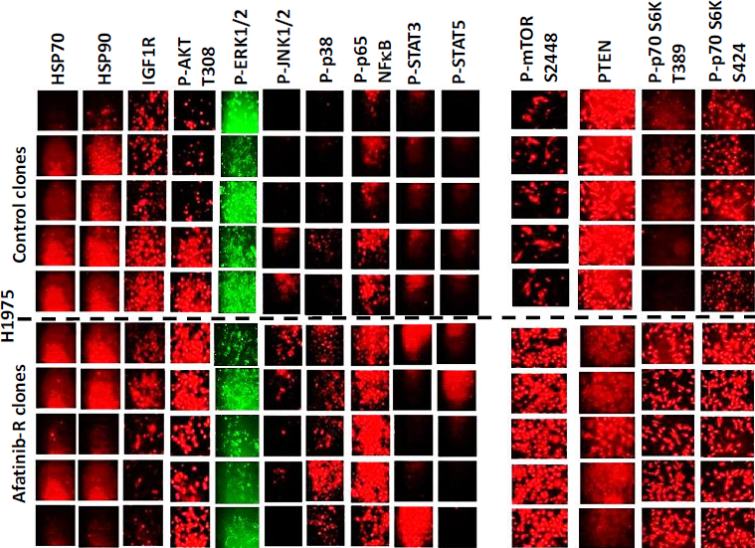
Clonal isolates of H1975 tumors from *in vivo* passaging and selection exhibit different biomarkers regardless of any drug exposure H1975 tumors (5 from vehicle control; 5 generated to become afatinib resistant) were dissociated and the clonal isolated cells grown *in vitro* as described in the Methods. Cells, 24 h after plating in the absence of any drugs were fixed *in situ* and immuno-fluorescence was performed to determine the expression of the indicated proteins: HSP70; HSP90; IGF1R; P-AKT T308; P-ERK1/2; P-JNK1/2; P-p38 MAPK; P-p65 NFκB; P-STAT3; P-STAT5; P-mTOR; PTEN; P-p70 S6K T389; P-p70 S6K S424.

**Figure 3:**
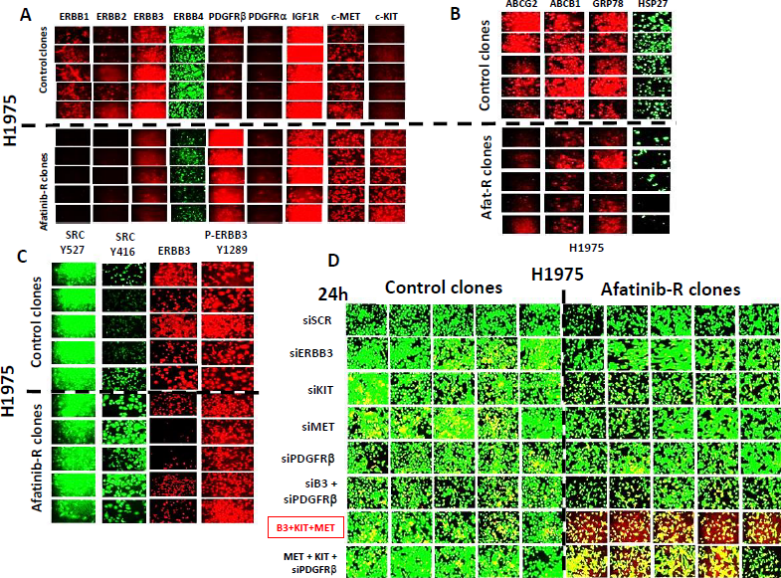
Afatinib resistant H1975 clones exhibit lower expression of ERBB1-4 and greater levels of c-MET, c-KIT and PDGFRβ; combined knock down of ERBB3, c-MET and c-KIT selectively kills afatinib resistant H1975 clones (**A**) H1975 tumors (5 from vehicle control; 5 generated to become afatinib resistant) were dissociated and the clonal isolated cells grown *in vitro* as described in the Methods. (**A**) Cells, 24 h after plating in the absence of any drugs were fixed *in situ* and immuno-fluorescence was performed to determine the expression of the indicated proteins: ERBB1, ERBB2, ERBB3, PDGFRβ, PDGFRα, IGF1R, c-MET and c-KIT. (**B**) Cells, 24 h after plating in the absence of any drugs were fixed *in situ* and immuno-fluorescence was performed to determine the expression of the indicated proteins: ABCG2, ABCB1, GRP78 and HSP27. (**C**) Cells, 24 h after plating in the absence of any drugs were fixed *in situ* and immuno-fluorescence was performed to determine the expression/phosphorylation of the indicated proteins: c-SRC Y527, c-SRC Y416, ERBB3, ERBB3 Y1289. (**D**) H1975 clones (5 from vehicle control; 5 generated to become afatinib resistant) were grown *in vitro* as described in the Methods. Cells were transfected with the indicated siRNA molecules to knock down the expression of growth factor receptors alone, in pairs or as a threesome. Twenty four h after transfection the viability of the tumor cells was assessed using a live/dead assay in the Hermes WiScan system. Green cells = alive; Yellow cells = red + green, dead but metabolically active; red cells = dead.

Afatinib resistant clones exhibited higher AKT T308, mTOR S2448, p70 S6K T389, p38 MAPK and p65 NFκB S536 phosphorylation and demonstrated a modest variable reduction in the phosphorylation of ERK1/2 and a substantial reduction in the total protein levels of the lipid phosphatase PTEN (Figure 2). Afatinib resistant H1975 clones had reduced expression of ERBB1, ERBB2, ERBB3 and ERBB4, and increased expression of c-KIT, c-MET and PDGFRβ (Figure 3A). ERBB1 and ERBB2 protein levels were reduced by > 80%; those of PDGFRβ increased by ∼275%; those of c-MET by ∼150%; and those of c-KIT by ∼400%. To our surprise expression of the drug efflux pumps ABCG2 and ABCB1 was reduced by ∼50% in afatinib resistant clones that correlated with reduced HSP27 and GRP78 levels (Figure 3B). The phosphorylation of c-SRC Y416 was increased and the phosphorylation of c-SRC Y527 was reduced in afatinib resistant clones. Although the expression of ERBB3 was considerably reduced in the afatinib resistant clones, the levels of ERBB3 Y1289 phosphorylation remained relatively constant suggesting that the stoichiometry of ERBB3 phosphorylation was profoundly increased in the afatinib resistant clones (Figure 3C). As we had observed so many changes in the expression and phosphorylation of growth factor receptors, we next performed a siRNA screen using control clones and afatinib resistant clones to determine which receptors, alone or in combination, were most responsible for the viability of the afatinib resistant cells. Selectively, in afatinib resistant clones, combined knock down of ERBB3, c-KIT and c-MET caused tumor cell death (Figure 3D).

**Figure 4:**
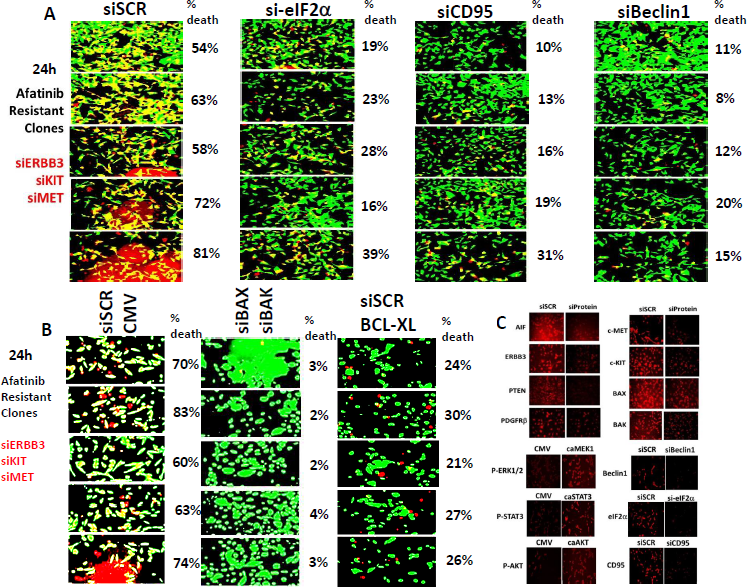
Afatinib resistant H1975 cell killing by knock down of [ERBB3 + c-KIT + c-MET] is partially reduced by knock down of eIF2α, CD95 or Beclin1 (**A**) H1975 afatinib resistant clones were transfected with siRNA molecules to knock down the expression of [ERBB3 + c-KIT + c-MET] combined. In parallel with this transfection cells were transfected with a scrambled siRNA or siRNA molecules to knock down expression of eIF2α, CD95 or Beclin1. Twenty four h after transfection cell viability was determined by live/dead assay in a Hermes WiScan machine. (**B**) H1975 afatinib resistant clones were transfected with siRNA molecules to knock down the expression of [ERBB3 + c-KIT + c-MET] combined. In parallel with this transfection cells were transfected with a scrambled siRNA; with siRNA molecules to knock down expression of BAX and BAK; or with a scrambled siRNA and a plasmid to express BCL-XL. Twenty four h after transfection cell viability was determined by live/dead assay in a Hermes WiScan machine. (**C**) Control immuno-fluorescence images 24 h after transfection showing the knock down of AIF, ERBB3, PTEN, PDGFRβ, BID, c-MET, c-KIT, BAX, and BAK.

Afatinib resistant tumor cell killing by [ERBB3 + c-KIT + c-MET] knock down was significantly, though only partially i.e. ∼70% reduction, reduced by knock down of eIF2α, CD95 or Beclin1 (Figure 4A, *p* < 0.05). The lethality of [ERBB3 + c-KIT + c-MET] knock down was reduced by combined knock down of [BAX + BAK] or of AIF (Figure 4B, data not shown). The lethality of [ERBB3 + c-KIT + c-MET] knock down was surprisingly only partially reduced by over-expression of BCL-XL. Control immuno-fluorescence data showing the knock downs of each of the proteins examined in the manuscript is presented in Figure 4C.

**Figure 5:**
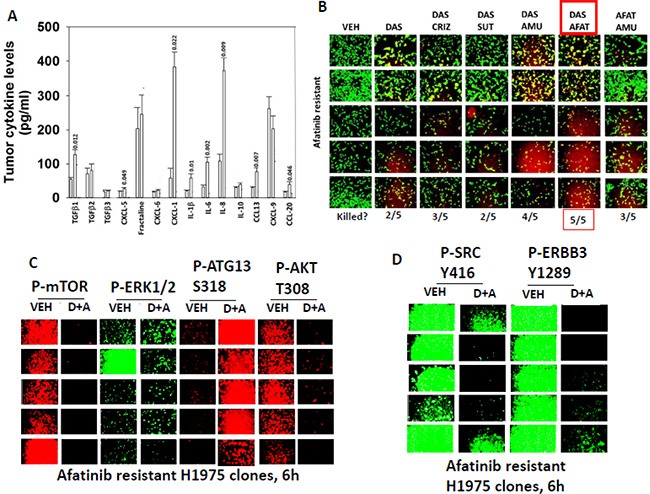
SRC signaling, as judged using dasatinib, plays a key survival regulatory role in afatinib H1975 cells (**A**) Animals carrying H1975 tumors were treated with afatinib. After the tumors re-grew animals were sacrificed and mouse plasma obtained. Clarified plasma was then subjected to multiplex assays as described in the Methods to detect the plasma levels of the indicated HUMAN cytokines using a Bio-Rad MAGPIX multiplex instrument (total 8 animals per condition, +/– SEM). The “*p*” values are listed above each bar. (**B**) H1975 clones were treated with vehicle control; afatinib (1 μM); dasatinib (1 μM); amuvatinib (1 μM); afatinib + dasatinib; dasatinib + amuvatinib; dasatinib + amuvatinib + afatinib. After 24 h the cells were treated with live/dead reagent and the amount of cell killing determined. (**C**) and (**D**) H1975 clones were treated with vehicle control; afatinib (1 μM); dasatinib (1 μM); amuvatinib (1 μM); afatinib + dasatinib; dasatinib + amuvatinib; dasatinib + amuvatinib + afatinib. After 6 h the cells were fixed *in situ* and immuno-fluorescence was performed to determine the expression of the indicated proteins and the phosphorylation of the indicated proteins.

Growth factor receptors such as ERBB3, c-KIT and c-MET are not only regulated by activating point mutations or deletions, or by receptor density, but also by their cognate ligands that can act in a paracrine/autocrine regulatory fashion. Compared to control clones, afatinib resistant H1975 clones/tumors expressed higher levels of TGFβ1, CXCL-1, IL-1β, IL-6, IL-8 and CCL-13. They did not however over-express heregulin family growth factors (ERBB3); stem cell factor and/or G-CSF (c-KIT); or hepatocyte growth factor (HGF) (Figure 5A, data not shown). Based on our knock down data with receptors, we next attempted to recapitulate our siRNA findings using clinically relevant drugs. Individual treatment of clones with the ERBB1/2/4 inhibitor afatinib; the c-KIT and c-MET inhibitor amuvatinib; the inhibitor of c-MET crizotinib; or the inhibitor of c-KIT, VEGF receptors and PDGF receptors sunitinib did not alter H1975 clone viability (Figure 5B, data not shown). Some afatinib resistant clones; 2 out of 5, were killed by single agent drug exposure using the c-SRC and c-KIT inhibitor dasatinib. Combined exposure of afatinib resistant clones to either [afatinib + dasatinib] (5/5 clones) or [amuvatinib + dasatinib] (4/5 clones) caused very high levels of tumor cell killing (Figure 5B). These drug combination effects on viability correlated with drug-induced: inactivation of AKT, mTOR, and ERK1/2, with increased phosphorylation of ATG13 S318; and with decreased phosphorylation of SRC Y416 and ERBB3 Y1289 (Figures 5C and 5D).

**Figure 6:**
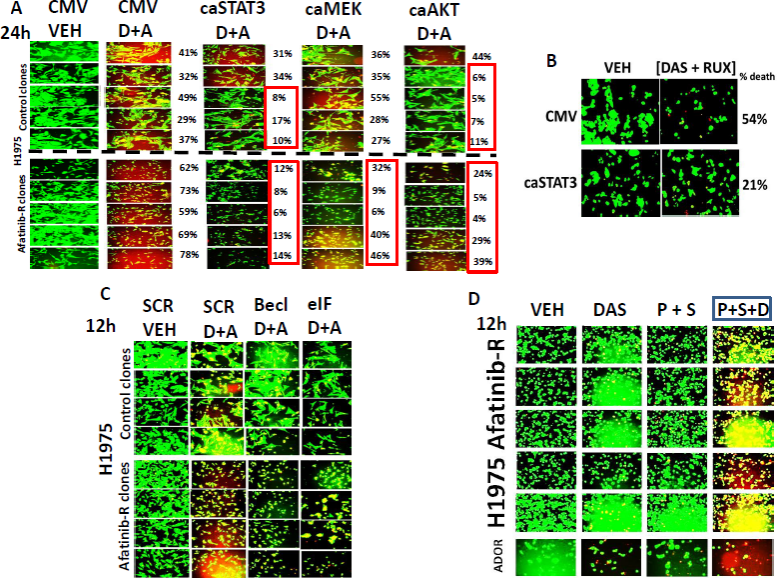
[Dasatinib + afatinib] kills afatinib resistant H1975 clones to a significantly greater extent than control clones and does so through inhibition of STAT3, MEK1 and AKT (**A**) H1975 control and afatinib resistant clones were transfected with an empty vector plasmid (CMV) or with plasmids to express: activated STAT3; activated MEK1; activated AKT. Twenty four h after transfection the cells were treated with vehicle control or were treated with [dasatinib 1 μM + afatinib 1 μM]. Twenty four h after drug exposure the viability of the tumor cells was assessed using a live/dead assay in the Hermes WiScan system. Green cells = alive; Yellow cells = red + green, dead but metabolically active; red cells = dead (+/− SEM). (**B**) A pool of the H1975 afatinib resistant cells were transfected with an empty vector plasmid (CMV) or with a plasmid to express activated STAT3. Twenty four h after transfection the cells were treated with vehicle control or were treated with [dasatinib 1 μM + ruxolitinib 1 μM]. Twenty four h after drug exposure the viability of the tumor cells was assessed using a live/dead assay in the Hermes WiScan system. (**C**) H1975 control and afatinib resistant clones were transfected with a scrambled siRNA molecule (siSCR) or siRNA molecules to knock down the expression of Beclin1 or eIF2 alpha. Twenty four h after transfection cells were treated with vehicle control or were treated with [dasatinib 1 μM + afatinib 1 μM]. Twenty four h after drug treatment the viability of the tumor cells was assessed using a live/dead assay in the Hermes WiScan system. Green cells = alive; Yellow cells = red + green, dead but metabolically active; red cells = dead (+/− SEM). (**D**) Afatinib resistant H1975 cells and the July 2015 NSCLC PDX isolate ADOR were treated with vehicle control; dasatinib (1 μM); pemetrexed (1.0 μM) + sorafenib (1.0 μM); or the three drugs together for 12 h. Twelve h after drug treatment the viability of the tumor cells was assessed using a live/dead assay in the Hermes WiScan system. Green cells = alive; Yellow cells = red + green, dead but metabolically active; red cells = dead.

In control H1975 clones; 3 out of 5 clones were protected from [dasatinib + afatinib] toxicity by expression of an activated form of STAT3, and 4 out of 5 clones were protected by expression of an activated form of AKT (Figure 6A). Expression of activated MEK1 was not protective in control clones. In afatinib resistant H1975 clones the expression of activated STAT3; or of activated MEK1; or of activated AKT all suppressed the lethality of [dasatinib + afatinib]. Thus the MEK1/2-ERK1/2 pathway represents a new key survival signal in afatinib resistant, but not control, clones. The cytokines CXCL-1 and CXCL-8 (IL-8) signal through 7-trans-membrane receptors whereas IL-6 signals through JAK1 and JAK2. As JAK1/2 regulate STAT3, and as activated STAT3 was shown to be protective we determined whether the clinically relevant JAK1/2 inhibitor ruxolitinib (Jakafi^®^) could interact with dasatinib to kill. Ruxolitinib and dasatinib interacted to kill afatinib resistant H1975 cells, an effect that was reduced by expression of activated STAT3 (Figure 6B). We again examined the impact of knocking down either Beclin1 or eIF2α on the viability of our H1975 clones, though now using [dasatinib + afatinib] treatment rather than siRNA knock down of receptors. In control clones knock down of Beclin1 had no impact on drug combination lethality, with knock down of eIF2α having an intermediate protective effect (Figure 6C). Afatinib resistant clones were more effectively killed by the drug combination (*p* < 0.05). Knock down of Beclin1 appeared to delay the killing caused by [dasatinib + afatinib] as judged by both drug treatment conditions having a similar rounded up pre-death morphology but that cells lacking Beclin1 had less uptake of ethidium bromide. Similar viability findings were also present in the eIF2α knock down cells.

In other studies we have shown that the drug combination of [pemetrexed + sorafenib] kills tumor cells *in vitro*, *in vivo*, and as was reported at the 2015 ASCO meeting, the phase I trial showed a 61% overall response rate in patients [7]. In follow up laboratory based studies we demonstrated that sorafenib-induced SRC activation was essential for drug combination killing; thus we would predict that the afatinib resistant H1975 clones with activated SRC would not be killed by this drug combination [8]. Afatinib resistant H1975 clones were killed by [pemetrexed + sorafenib], an effect that was magnified by dasatinib (Figure 6D, data not shown).

## DISCUSSION

The present studies were designed to examine the biology of H1975 tumors that had been treated with afatinib until the tumor volume was zero, and then permitted to re-grow as afatinib resistant tumors. In recent months there have been two other studies published examining the biology of H1975 cells being made resistant to afatinib [9, 10]. One study stated that IGF1R signaling was responsible for afatinib resistance whereas the other stated that activation of the AKT and ERK1/2 pathways played a role. Several years ago another group argued that STAT3 signaling was a key player in H1975 afatinib resistance [11]. In all three prior studies, H1975 cells *in vitro* were made afatinib resistant by prolonged treatment of the cells with increasing concentrations of afatinib, in one case up to 10 μM of the drug. The present studies represent a more clinically relevant model in which H1975 tumor cells as growing tumors were transiently treated with high concentrations of afatinib, the tumors allowed to completely regress, and then re-grow, before harvesting for *in vitro* analyses.

Prior to any genomic analyses by the fee-for-service MCVH Department of Pathology laboratory, our a priori prediction was that we would observe at least one new ‘hot-spot’ mutation in a pivotal upstream signal transduction pathway which would protect against cell death e.g. N-/K-RAS. It was thus a surprise when our screen of multiple hotspot mutation sites revealed no mutational changes whatsoever. Because of our fortunate access to a Hermes WiScan machine, we already had a large number of data sets examining the phosphorylation and expression of various proteins in the resistant and control H1975 clones; proteins we knew and in part strongly believed would be involved in any adaptation process in the tumor cell becoming afatinib resistant. The most obvious initial observation when the data was collated was that ERBB family receptor expression had declined precipitously and the expression of c-KIT, c-MET and PDGFRβ were elevated. At the intracellular level we noted that the total expression of PTEN had declined which correlated with increased phosphorylation of multiple PI3K pathway effectors downstream of PTEN including AKT, mTOR and p70 S6K. As discussed in the companion manuscript, the stoichiometry of PTEN S380 phosphorylation was increased in afatinib resistant H1975 clones, which will result in a further derogation of PTEN function. It was then discovered that c-SRC was activated in afatinib resistant cells as judged by increased Y416 and decreased Y527 phosphorylation; this was associated with a very high stoichiometry of ERBB3 Y1289 phosphorylation. Based on our data and logic, we knocked down the most probable growth factor receptors alone or in combination and discovered that combined knock down of ERBB3, c-KIT and c-MET was selectively profoundly toxic to afatinib resistant H1975 cells, but not to control H1975 cells. The combination of PDGFRβ, c-KIT and c-MET knock down was also found to have a significant intermediate level of afatinib resistant cell specific killing.

The commonality for both three receptor combinations are c-KIT and c-MET; with wild type c-KIT only expressed in the afatinib resistant cells. The proto-oncogene c-KIT, also known as the stem cell growth factor receptor, binds to stem cell factor and is also indirectly activated by G-CSF. Dasatinib is a tyrosine kinase inhibitor with clinically validated activity against BCR-ABL, c-SRC and c-KIT. Thus, based on the fact we observed activation of c-SRC and over-expression of c-KIT in our afatinib resistant clones, we chose this drug as part of our screening to determine what clinically relevant drugs might combine to kill these clones. The question we then posited was what would be the most efficacious second drug to combine with dasatinib based on the protein molecular fingerprint in the afatinib resistant clones. We tested crizotinib, an inhibitor of c-MET; sunitinib, an inhibitor of PDGF receptors, VEGF receptors and c-KIT; and amuvatinib, an inhibitor of c-KIT, PDGF receptors and FLT3; alongside afatinib and dasatinib. To our surprise, considering that the expression of ERBB1/2/4 had been significantly reduced, afatinib combined with dasatinib to kill 5 out of 5 clones. These data argue that residual signaling from ERBB1/2/4 still plays a vital role in regulating H1975 viability.

We also observed increased expression of cytokines and growth factors in the afatinib resistant H1975 clones though none of those factors that were elevated are capable of activating ERBB3, c-KIT or c-MET. Due to cessation of laboratory funding by the VCU Massey Cancer Center for our studies, future experiments examining the roles of CXCL-1 and CXCL-8 in our afatinib resistance system, through 7-trans-membrane receptors and hetero-trimeric G proteins, have been cancelled. However, we were able to perform a short proscribed series of studies examining the role of IL-6 signaling via JAK1/2-STAT3 on afatinib resistant H1975 cell viability using the clinically relevant drug ruxolitinib. Ruxolitinib enhanced dasatinib lethality in the afatinib resistant clones, an effect that was abolished by expression of activated STAT3 (Unpublished results). Thus we were able to validate at least one paracrine survival pathway in our system from IL-6, through its receptor and JAK1/2, to STAT3, to the regulation of protective factor expression such as BCL-XL. As our H1975 tumors were grown in athymic mice that only lack T cells, it is possible that the source of the IL-6 is from a hematological / marrow stem cell source rather than the tumor cells themselves.

One noticeable difference in the killing of the afatinib H1975 tumor cells between [siERBB3 + si-c-MET + si-c-KIT] and [dasatinib + afatinib] was the relative impact of blocking autophagosome formation and of blocking eIF2α-dependent endoplasmic reticulum stress signaling. Killing the afatinib resistant clones by receptor knock down provided strong evidence that death receptor signaling was playing a key upstream role in causing tumor cell death, and that downstream both endoplasmic stress signaling through eIF2α and autophagosome formation through Beclin1 were essential mediators of this death signal. That BAX/BAK were essential for killing but that BCL-XL was only partially protective suggests the possibility of a necroptotic form of cell killing. In contrast to the genetic data, [dasatinib + afatinib] exposure killed cells in a manner that was only weakly blunted by knock down of eIF2α or Beclin1. Clearly, as dasatinib likely has partial inhibitory effects on many tyrosine kinases in addition to c-SRC and c-KIT that are rarely discussed in the literature, the drug combination in contrast to the molecular approach can recruit “additional pathways to death.” As heterozygous deletion of Beclin1 is well known to be one mechanism used during the development of cancer and for resistance to some traditional chemotherapeutic modalities, our data show that by attacking multiple survival pathways simultaneously, i.e. not just Beclin1, we gain an advantage over any therapeutic intervention that is highly specific e.g. AZD9291 [12, 13]. It is hoped that colleagues elsewhere will be able to perform future studies to define whether and how CXCL-1 and CXCL-8 signal to protect cells or studies to define all of the necroptotic pathways being engaged by either receptor knock down or [dasatinib + afatinib / ruxolitinib] are unlikely to proceed.

At present there is a clinical trial open at the H. Lee Moffitt Cancer Center and Research Institute combining afatinib and dasatinib in patients who are initially presenting with a non-small cell lung cancer that harbors a mutated active form of ERBB1 (NCT01999985). A research group at the Moffitt Cancer Center using mass spectrometric and other high end instrument methodologies has shown, as a part of their studies, that afatinib and dasatinib combine to kill lung cancer cells expressing a mutated active ERBB1, though no actual studies were performed to revert afatinib resistance and in an ERBB1 T790M L858R double mutated receptor cell line [14]. Collectively our data argues that an additional exploratory phase I trial combining afatinib and dasatinib should be proposed in NSCLC patients who have failed single agent afatinib therapy. And, in addition, and based on the results of the on-going phase I trial, that the addition of the JAK1/2 inhibitor ruxolitinib in a pulsatile fashion to [dasatinib + afatinib] may provide additional tumor control and patient benefit.

## MATERIALS AND METHODS

### Materials

Afatinib, dasatinib, pazopanib, amuvatinib, crizotinib, sunitinib, sorafenib tosylate and copanlisib were purchased from Selleckchem (Houston, TX). Trypsin-EDTA, DMEM, RPMI, penicillin-streptomycin were purchased from GIBCOBRL (GIBCOBRL Life Technologies, Grand Island, NY). Cells were purchased from the ATCC and were not further validated beyond that claimed by ATCC. Cells were re-purchased every ∼6 months. Commercially available validated short hairpin RNA molecules to knock down RNA / protein levels were from Qiagen (Valencia, CA) or were supplied by collaborators. Reagents and performance of experimental procedures were described in [15–18].

### Methods

### Culture and *in vitro* exposure of cells to drugs

All cell lines were cultured at 37°C (5% (v/v CO_2_) *in vitro* using RPMI supplemented with dialyzed 5% (v/v) fetal calf serum and 10% (v/v) Non-essential amino acids. *In vitro* drug treatments were from 100 mM stock solutions of each drug and the maximal concentration of Vehicle (DMSO) in media was 0.02% (v/v). Cells were not cultured in reduced serum media during any study in this manuscript.

### Transfection of cells with siRNA or with plasmids

For Plasmids: Cells were plated and 24 h after plating, transfected. Plasmids expressing a specific mRNA (or siRNA) or appropriate vector control plasmid DNA was diluted in 50 μl serum-free and antibiotic-free medium (1 portion for each sample). Concurrently, 2 μl Lipofectamine 2000 (Invitrogen), was diluted into 50 μl of serum-free and antibiotic-free medium (1 portion for each sample). Diluted DNA was added to the diluted Lipofectamine 2000 for each sample and incubated at room temperature for 30 min. This mixture was added to each well/dish of cells containing 200 μl serum-free and antibiotic-free medium for a total volume of 300 μl, and the cells were incubated for 4 h at 37°C. An equal volume of 2× medium was then added to each well. Cells were incubated for 24 h, then treated with drugs.

Transfection for siRNA: Cells from a fresh culture growing in log phase as described above, and 24 h after plating transfected. Prior to transfection, the medium was aspirated and serum-free medium was added to each plate. For transfection, 10 nM of the annealed siRNA, the positive sense control doubled stranded siRNA targeting GAPDH or the negative control (a “scrambled” sequence with no significant homology to any known gene sequences from mouse, rat or human cell lines) were used. Ten nM siRNA (scrambled or experimental) was diluted in serum-free media. Four μl Hiperfect (Qiagen) was added to this mixture and the solution was mixed by pipetting up and down several times. This solution was incubated at room temp for 10 min, then added drop-wise to each dish. The medium in each dish was swirled gently to mix, then incubated at 37°C for 2 h. Serum-containing medium was added to each plate, and cells were incubated at 37°C for 24 h before then treated with drugs (0–24 h). Additional immuno-fluorescence/live-dead analyses were performed at the indicated time points.

### Animal studies (lung cancer)

For studies to generate afatinib resistant H1975 cells, athymic nude mice (∼20 g) were injected with 1 × 10^7^ H1975 cells into their rear flank. Tumors were permitted to form for 7 days with tumors at that time exhibiting a mean volume of 25–50 mm^3^. Athymic mice were treated by oral gavage twice every day BID for four days with vehicle (Cremophore) or with afatinib (50 mg/kg). After cessation of drug treatment tumors treated twice daily with afatinib showed a reduction in tumor volume of all treated tumors to 0 mm^3^ for approximately 7 days after which tumors began to slowly re-grow. Recurrent tumors were isolated twenty five days after they exhibited the initial re-formation of a small tumor, when they had a volume of ∼500 mm^3^, portions were either snap-frozen or were digested to release individual tumor cells, and cells from each tumor clone maintained separately i.e. we generated 5 control / vehicle treated clones from 5 separate tumors and we generated 5 afatinib-resistant clones from 5 separate tumors. Control treated tumors were also isolated when they had a volume of ∼500 mm^3^. Of significant note for clonal characterization, the isolated afatinib treated tumor clones' cells were only growth inhibited by afatinib when cultured *in vitro* with daily supplementation at concentrations >> 2 μM, and as such these afatinib resistant cells were routinely passaged in a pulsatile fashion between experiments in growth media containing only 1 μM afatinib to maintain the afatinib resistant phenotype but not to promote further selective pressure on drug resistance and thus also did not cause selection of surviving clones due to non-ERBB1/2/4 off-target effects.

### Multiplex assays for cytokine expression

A Bio-Rad MAGPIX instrument with associated software was purchased from Bio-Rad. The following Bio-Plex assay plates were used in our assays of mouse tumor tissue: PRO Mouse Cyto 23-PLEX (M60009RDPD); PRO TGF-B 3-PLEX (171W4001M); Mouse Cyto STD GRP II 9-PLEX (171I60001). Mouse tumor tissue was assayed according to the instructions provided by Bio-Rad and with Bio-Rad technical assistance.

### Detection of cell viability, protein expression and protein phosphorylation by immuno-fluorescence using a Hermes WiScan machine

http://www.idea-bio.com/, Cells (4 × 10^3^) are plated into each well of a 96 well plate, and cells permitted to attach and grow for the next 18 h. Based on the experiment, after 18 h, cells are then either genetically manipulated, or are treated with drugs. For genetic manipulation, cells are transfected with plasmids or siRNA molecules and incubated for an additional 24 h. Cells are treated with vehicle control or with drugs at the indicated final concentrations, alone or in combination. Cells are then isolated for processing at various times following drug exposure. The 96 well plate is centrifuged / cyto-spun to associate dead cells (for live-dead assays) with the base of each well. For live dead assays, after centrifugation, the media is removed and cells treated with live-dead reagent (Thermo Fisher Scientific, Waltham MA) and after 10 min this is removed and the cells in each well are visualized in the Hermes instrument at 10× magnification. Green cells = viable; yellow/red cells = dying/dead. The numbers of viable and dead cells were counted manually from three images taken from each well combined with data from another two wells of separately treated cells (i.e. the data is the mean cell dead from 9 data points from three separate exposures). For immuno-fluorescence studies, after centrifugation, the media is removed and cells are fixed in place and permeabilized using ice cold PBS containing 0.4% paraformaldehyde and 0.5% Triton X-100. After 30 min the cells are washed three times with ice cold PBS and cells are pre-blocked with rat serum for 3 h. Cells are then incubated with a primary antibody to detect the expression/phosphorylation of a protein (usually at 1:100 dilution from a commercial vendor) overnight at 37°C. Cells are washed three times with PBS followed by application of the secondary antibody containing an associated fluorescent red or green chemical tag. After 3 h of incubation the antibody is removed and the cells washed again. The cells are visualized at either 10× or 60× in the Hermes machine for imaging assessments. All immunofluorescent images for each individual protein/phospho-protein are taken using the identical machine settings so that the levels of signal in each image can be directly compared to the level of signal in the cells treated with drugs. Similarly, for presentation, the enhancement of image brightness/contrast using PhotoShop CS6 is simultaneously performed for each individual set of protein/phospho-protein to permit direct comparison of the image intensity between treatments.

### Data analysis

Comparison of the effects of various treatments was performed using one way analysis of variance and a two tailed Student's *t*-test. Experiments shown are the means of multiple individual points from multiple experiments (± SEM).

## Abbreviations

ERK: extracellular regulated kinase
MEK: mitogen activated extracellular regulated kinase
PI3K: phosphatidyl inositol 3 kinase
ca: constitutively active
dn: dominant negative
PTX: pemetrexed
ER: endoplasmic reticulum
mTOR: mammalian target of rapamycin
MAPK: mitogen activated protein kinase
PTEN: phosphatase and tensin homologue on chromosome ten
ROS: reactive oxygen species
CMV: empty vector plasmid or virus
si: small interfering
SCR: scrambled
IP: immunoprecipitation
Ad: adenovirus
VEH: vehicle
